# LINC00319 promotes cancer stem cell-like properties in laryngeal squamous cell carcinoma via E2F1-mediated upregulation of HMGB3

**DOI:** 10.1038/s12276-021-00647-2

**Published:** 2021-08-18

**Authors:** Linlin Yuan, Xiufen Tian, Yanfei Zhang, Xinhui Huang, Qing Li, Wencai Li, Shenglei Li

**Affiliations:** 1 Department of Otorhinolaryngology Head and Neck Surgery, The First Affiliated Hospital of Zhengzhou University, 450052 Zhengzhou, People’s Republic of China; 2 Department of Pathology, The Third Affiliated Hospital of Soochow University (Changzhou City No. 1 People’s Hospital), 215006 Changzhou, People’s Republic of China

**Keywords:** Cancer, Diseases

## Abstract

Laryngeal squamous cell carcinoma (LSCC) is one of the most common subtypes of head and neck malignancies worldwide. Long intervening/intergenic noncoding RNAs (LINCRNAs) have been recently implicated in various biological processes that take place in the setting of laryngeal cancer, but the regulatory role of LINC00319 in LSCC remains largely unknown. The current study aimed to elucidate the regulatory effect of LINC00319 on the development and progression of LSCC via high-mobility group box 3 (HMGB3). Microarray-based analysis was initially conducted to identify differentially expressed long noncoding RNAs, after which the expression of LINC00319 and HMGB3 in LSCC tissues and cells was determined accordingly. CD133^+^CD144^+^ TU177 cells were subsequently isolated and transfected with LINC00319 overexpression vector (oe-LINC00319), short hairpin RNA (sh)-LINC00319, sh-HMGB3, sh-E2F transcription factor 1 (E2F1), and oe-E2F1, as well as their corresponding controls. The proliferative, invasion, self-renewal, and tumorigenic abilities of CD133^+^CD144^+^ TU177 cells were then evaluated. Our in vitro findings were further confirmed following subcutaneous injection of cells expressing the corresponding plasmids into nude mice. LINC00319 and HMGB3 expressions were elevated in LSCC cells and tissues. LINC00319 increased HMGB3 expression by recruiting E2F1. Furthermore, the stimulatory role of LINC00319 on the proliferation, invasion, self-renewal ability, and tumorigenicity of CD133^+^CD144^+^ TU177 cells was achieved by upregulating HMGB3 via recruitment of E2F1. The in vitro findings were also confirmed by in vivo experiments. Taken together, these data show that downregulating LINC00319 in CD133^+^CD144^+^ TU177 cells may serve as a potential anticancer regimen by inhibiting the proliferation and invasion of cancer stem cells in LSCC.

## Cancer: An RNA promotes throat cancer progression

The identification of a cellular RNA that drives tumor proliferation and invasion could lead to new treatments for a common form of throat cancer that often presents with a grim prognosis. With early diagnosis, laryngeal squamous cell carcinoma (LSCC) can be treated effectively, but patients with advanced or recurrent disease have a 5-year survival rate of just 50%. Researchers led by Shenglei Li and Wencai Li at Zhengzhou University in China have found that increased production of an RNA known as LINC00319 is linked with worse outcomes for LSCC patients. This RNA does not encode a protein, but instead regulates expression of other genes that allow reservoirs of ‘cancer stem cells’ associated with tumor progression to flourish. Based on these findings, the researchers propose that drugs targeting LINC00319-regulated cellular processes could offer useful LSCC therapeutics.

## Introduction

Laryngeal cancer remains one of the most common malignancies afflicting people across the globe and is often associated with a high incidence rate and mortality^1^. Laryngeal squamous cell carcinoma (LSCC) represents the greater majority of laryngeal cancers, ranking as the second most common tumor occurring in the head and neck region worldwide with ~151,000 new cases occurring on an annual basis^2^. LSCC often manifests as persistent pain, cough, bad breath, and difficulty swallowing connected to the larynx^3^. Existing treatment approaches for early laryngeal cancer comprise surgery, radiotherapy, or chemotherapy either alone or in combination^1^. Despite encouraging developments from an LSCC treatment perspective, patient outcomes remain unsatisfactory, particularly among those diagnosed at an advanced stage or with recurrent disease, where the 5-year survival is <50%^4^. Importantly, cancer stem cells (CSCs), a small subpopulation of cancer cells, have been found to be of significance with respect to self-renewal and tumorigenicity and are regarded as the major driving force in tumor progression and metastasis after traditional treatment^5^. Thus, it is imperative that we continue to identify new treatments for LSCC based on CSCs.

Long intervening noncoding RNAs (LINC findings highlight theNAs), which are transcribed from thousands of loci in mammalian genomes, have been highlighted as crucial factors involved in the regulation of gene expression as well as the mediation of various cellular processes^6^. Located on chromosome 21q22.3, long noncoding RNA (lncRNA) LINC00319 has a transcript length of 2901 nucleotides^7^. Existing literature has suggested that overexpression of LINC00319 is associated with poor patient prognosis and linked to cell proliferation and invasion in cutaneous squamous cell carcinoma^8^. Previous reports have suggested that upregulation of LINC00339 can enhance cell proliferation and invasion in LSCC^9^. All these previous reports implicate LINC00319 as an oncogene. However, the underlying biological mechanism of LINC00319 in LSCC CSCs remains poorly understood.

As an X-linked member of the high-mobility group superfamily of HMG proteins, high-mobility group box 3 protein (HMGB3) has been linked with the cell cycle by activating the endogenous cyclin A gene, which promotes tumor growth in gastric cancer^10^. Silencing HMGB3 has been reported to suppress cell proliferation and migration while influencing chemosensitivity in gastric cancer^11^. However, to our knowledge, no evidence reporting the role of HMGB3 in LSCC has ever been made available. E2F transcription factor 1 (E2F1) protein represents a key member of the E2F family of transcription factors and plays a primary role in mediating cell cycle distribution and the function of tumor suppressors, while it is also well known to be a target of transforming proteins of small DNA tumor viruses^12^. The E2F proteins contain several evolutionarily conserved domains detected in most members of the family, including a DNA-binding domain determining interaction with differentiation-mediated transcription factor proteins, a transactivation domain enriched in acidic amino acids, and a tumor suppressor association domain embedded within the transactivation domain (http://genome-asia.ucsc.edu/cgi-bin/hgGene?hgg_gene=ENST00000343380.6&hgg_prot=uc002wzu.6&hgg_chrom=chr20&hgg_start=33675476&hgg_end=33686385&hgg_type=knownGene&db=hg38&hgsid=744961287_w8VMXYhWoR4pZAGuZfAOusN5AXD6). The transcription factor E2F1 has also been highlighted as a tumor-associated gene based on its role in the cell cycle pathway, with targets of E2F1 reported to play an important role in the progression of LSCC^13^. One of the lncRNA whose functions involve the recruitment of transcription factors at the gene promoter region of E2F1 has been identified^14^. LncRNA metastasis-associated lung adenocarcinoma transcript 1 has been reported to share a correlation with E2F1 in terms of cell development and tumorigenesis^15^. Previous evidence has demonstrated that lncRNA E2F1-regulated inhibitor of cell death associates with AT-rich interactive domain 3A via E2F1 to promote osteosarcoma progression ^16^. These findings highlight the role of lncRNAs in various cancers via the regulation of E2F1. Hence, we suspect that LINC00319 plays a role in LSCC via manipulation of E2F1. In addition, recent evidence has demonstrated that elevated expression of HMGB2 in various human cancers can be suppressed by the small heterodimer partner via recruitment and activation of E2F1 ^17^. Given the aforementioned exploration of the literature, we hypothesized that LINC00319 is involved in mediating the proliferation, invasion, and self-renewal ability of CSCs in vitro and tumorigenesis through its interaction with HMGB3 and E2F1 in LSCC.

## Materials and methods

### Ethics sta**t**ement

The current study was performed with the approval of the Ethics Committee of The First Affiliated Hospital of Zhengzhou University (approval number: 2017034). Written informed consent was obtained from each patient prior to enrollment in the study. All experimental procedures involving animals were performed strictly for medical research purposes and were approved by the Animal Ethics Committee of The First Affiliated Hospital of Zhengzhou University (approval number: 2017A-029).

### **B**ioinformatics analysis

The Gene Expression Omnibus (https://www.ncbi.nlm.nih.gov/geo/) database was used to obtain the microarray data of LSCC and annotation probe files. Background correction and normalization of microarray data were conducted by using Affy in R^18^. A linear model-empirical Bayesian statistics method in the Limma software and traditional *t* test were used to screen out differentially expressed genes on a transcriptional level^19^ with |log FoldChange| > 2 and *p* < 0.05 set as the screening criteria.

### Clinical sample and cell culture

A total of 87 pairs of cold fresh clinical LSCC samples and adjacent tissues were obtained from patients with LSCC at The First Affiliated Hospital of Zhengzhou University between March 2017 and May 2018. A total of four LSCC cell lines, TU177, AMC-HN-8, TU212, and TU686, and immortalized human epidermal HaCaT cell lines were purchased from American Type Culture Collection (Rockville, MD, USA). The cells were cultured in Dulbecco’s modified Eagle’s medium (DMEM) (Gibco, Grand Island, NY, USA) containing 10% fetal bovine serum (FBS), 100 mg/mL streptomycin and 100 U/mL penicillin (90% high-glucose DMEM + 10% FBS + 1% penicillin–streptomycin solution) in an incubator with 5% CO_2_ and 95% humidity at 37 °C. Cell passage was conducted upon the cultures reaching 80% confluence.

### Plasmid delivery

Following detachment, LSCC cells were seeded into a six-well plate at a density of 1 × 10^6^ cells/well and cultured for 24 h. Upon reaching a cell confluence of ~75%, cell transfection was performed in accordance with the manual of the Lipofectamine 2000 kit (Invitrogen, Carlsbad, CA, USA). LSCC cells were treated with the following plasmids: LINC00319 overexpression (oe-LINC00319), short hairpin (sh) RNA against LINC00319 (sh-LINC00319), oe-LINC00319 + sh-HMGB3, oe-LINC00319 + sh-E2F1, or oe-LINC00319 + oe-E2F1 + sh-HMGB3, in addition to their corresponding negative controls (NCs), oe-NC, sh-NC, oe-NC + sh-NC, oe-LINC00319 + sh-NC, and oe-LINC00319 + oe-E2F1 + sh-NC. Opti-MEM (250 μL, Gibco) was utilized to dilute 4 μg of target plasmids and 10 μL Lipofectamine 2000. After standing at room temperature for 5 min, the reagents were mixed and allowed to stand at room temperature for 20 min. A mixture of Lipofectamine 2000 and plasmids was then added to different wells and gently mixed for an 8-h period. The medium was subsequently replaced with the fresh medium. The cells were collected 48 h later for subsequent experiments. All plasmids were purchased from Dharmacon (Lafayette, CO, USA).

### Flow cytometry for cell sorting

TU177 cells were dispersed into a single-cell suspension, and the cell density was adjusted to 1 × 10^6^ cells/mL. The cells were suspended in 100 μL culture medium with 10 μL phycoerythrin-labeled CD133 antibody (Milteny, Bergisch Gladbach, Germany) and 10 μL fluorescein isothiocyanate (FITC)-labeled CD144 antibody (Milteny), followed by a 30-min incubation at room temperature in the dark. After two washes with phosphate-buffered saline (PBS), CD133^+^CD144^+^ TU177 cells were obtained by sorting.

### Immunocytochemistry

Slides of CD133^+^CD144^+^ TU177 cell subpopulations were prepared, fixed in 4% paraformaldehyde solution for 15 min at room temperature, washed three times with PBS, and blocked with 10% goat serum. A rabbit anti-human antibody against CD133 (ab19898, 1:1000) was added, after which a mouse anti-human antibody against CD144 (ab19898, 1:1000) was added and incubated with the sample at 4 °C overnight. The samples were subsequently incubated with tetramethylrhodamine-conjugated goat anti-rabbit immunoglobulin G (IgG) H&L (ab6718, 1:1000) and FITC-conjugated goat anti-mouse IgG H&L (ab6785, 1:1000) secondary antibodies for 45 min, washed with PBS three times, and sealed with Vectashield (Vector Laboratories Inc., Burlingame, CA, USA). All aforementioned antibodies were purchased from Abcam Inc. (Cambridge, MA, USA). A laser scanning confocal microscope (Olympus, Tokyo, Japan) was employed for observation and photography of the processed cell slides.

### Reverse transcription-quantitative polymerase chain reaction (RT-qPCR)

Total RNA was extracted from the tissues and cells using TRIzol (15596026, Invitrogen). RNA was reverse transcribed into complementary DNA using an RT kit (RR047A, Takara, Japan), and the samples were added to components of an SYBR Premix EX Taq kit (RR420A, Takara). RT-qPCR was conducted in a real-time fluorescent qPCR instrument (ABI7500, ABI, Foster City, CA, USA). Triplicate wells were set for each sample. All primers were synthesized by Sangon Biotech Co., Ltd (Shanghai, China) (Table S1). The Ct value was recorded with glyceraldehyde-3-phosphate dehydrogenase (GAPDH) as the internal reference. The fold changes between the experimental group and the control group were calculated using relative quantification 2^−ΔΔCt^.

### Fluorescence in situ hybridization (FISH)

FISH was conducted to detect the subcellular localization of LINC00319 according to the instructions of the Ribo^TM^ lncRNA FISH Probe Mix (Red) (C10920, RiboBio Biological Technology Company, Guangzhou, Guangdong, China). A cell slide was placed in each well of the 24-well plate at 6 × 10^4^ cells per well. Upon reaching 60–70% confluence, the cell cultures were fixed in 4% paraformaldehyde solution for 10 min, added to 1 mL precooled PBS containing 0.5% Triton X-100 in each well for 5 min at 4 °C, and washed accordingly. Each well was subsequently blocked for 30 min using 200 μL prehybridization solution and incubated with hybridization solution containing anti-LINC00319 nucleotide probes (Genecreate Biotech Co., Ltd, Wuhan, Hubei, China) overnight at 37 °C. The cells were then washed with 42 °C solution I (4× saline sodium citrate [SSC], 0.1% Tween-20), solution II (2× SSC), solution III (1× SSC), and 1× PBS three times (each for 5 min), followed by staining with 4′,6-diamidino-2-phenylindole (1:800) for 5 min before they were finally sealed with nail polish. A total of five visual fields were randomly selected for observation under a fluorescence microscope (Olympus), after which photos were taken.

### Immunohistochemistry

LSCC tissues were fixed in 4% paraformaldehyde solution for 12 h and dehydrated using a gradient series of ethanol in ascending concentrations. The tissue blocks were then placed into a mixture comprising equal volumes of absolute alcohol and xylene for 0.5 h and soaked twice in xylene for 20 min each. The paraffin-embedded blocks were subsequently cut into sections that were sequentially rinsed with 3% H_2_O_2_ for 10 min and blocked with a blocking solution containing 10% goat serum (Cwbiotech Co., Ltd, Beijing, China) at room temperature for 20 min. Primary antibody to HMGB3 protein (50 μL, ab75782, 1:100, Abcam) was added and incubated with the sample for 1 h at room temperature, followed by the addition of 50 μL secondary goat anti-mouse antibody to IgG (ab6721, 1:1000, Abcam) for 1 h at room temperature. Streptavidin-conjugated peroxidase was then added to react with the sample at 37 °C for 30 min. The samples were visualized using diaminobenzidine for 5–10 min, after which the reaction was terminated by washing with running water for 10 min, followed by counterstaining with hematoxylin and differentiation with hydrochloric alcohol. Following conventional dehydration, clearing, and sealing, the samples were observed under a fluorescence microscope.

### RNA immunoprecipitation (RIP)

A RIP assay kit (Millipore, MA, USA) was used to detect binding between LINC00319 and the transcription factor E2F1^20^. A percentage of the cell extract was used as the input, while the remaining portion was incubated with antibodies and magnetic beads for pulldown. The immunoprecipitated complexes were eluted and resuspended in 100 μL RIP Wash Buffer. The sample was placed onto a magnetic column to collect magnetic bead–protein complexes. RNA was extracted from the samples that were first treated with proteinase K for PCR analysis. An antibody against E2F1 (ab179445, 1:100, Abcam) was introduced for a 30-min incubation at room temperature with IgG (ab172730, 1:100, Abcam) regarded as the NC.

### Western blot analysis

Radioimmunoprecipitation assay lysis buffer containing phenylmethanesulfonyl fluoride was employed to extract protein from the tissues and cells. A bicinchoninic acid assay kit was used to identify the protein concentration. The proteins were separated by sodium dodecyl sulfate-polyacrylamide gel electrophoresis and transferred onto a polyvinylidene fluoride membrane, which was then blocked with 5% dried skim milk at room temperature for 1 h. The membrane was incubated with diluted primary rabbit antibody against HMGB3 (ab75782, 1:2500) with GAPDH antibody (ab9485, 1:2500) as the internal reference. The membrane was subsequently incubated with horseradish peroxidase-conjugated secondary goat anti-rabbit antibody to IgG H&L (ab97051, 1:2000). All the mentioned antibodies were provided by Abcam. A Bio-Rad picture analysis system (Bio-Rad, CA, USA) was used to capture pictures, and Quantity One v4.6.2 software was employed for analysis. The relative protein expression is presented as the ratio of the gray value of the target bands to that of the GAPDH band. The experiment was repeated three times with the average gray value obtained accordingly.

### Dual-luciferase reporter gene assay

Luciferase reporter plasmids (HMGB3-2Kb) were co-transfected with oe-NC, oe-LINC00319, sh-NC, and sh-LINC00319 into CSCs to examine the influence of LINC00319 on the HMGB3 promoter. After 48 h, the cells were collected and lysed. A luciferase detection assay kit (K801-200, Biovision, CA, USA) was applied, after which a dual-luciferase reporter gene analysis system (Promega, Madison, WI, USA) was introduced. Renilla luciferase was used as the internal reference. The activity of the target reporter gene was calculated as the ratio of the relative light unit (RLU) value of firefly luciferase to that of Renilla luciferase. Next, oe-NC, oe-LINC00319, sh-NC, and sh-LINC00319 were co-transfected with the E2F1 luciferase reporter vector into TU177 stem cells to detect the binding between LINC00319 and E2F1 with Renilla luciferase as the internal reference. The cells were lysed at 48 h after transfection. A luciferase detection kit (K801-200, Biovision) and dual-luciferase reporter gene analysis system (Promega) were employed to determine the luciferase activity. The activity of the target reporter gene was regarded as the ratio between the RLU value of firefly luciferase and that of Renilla luciferase.

Our data suggested that E2F1 was likely to bind to three sites in the *HMGB3* gene based on the predictions provided by UCSC (http://genome.ucsc.edu/) and JASPAR (http://jaspar.genereg.net/). A dual-luciferase reporter gene vector containing truncated or mutant (MUT) sequences of each predictive binding site was reconstructed and subsequently co-transfected with E2F1 into CSCs for a dual-luciferase reporter gene assay to verify the specific binding between E2F1 and HMGB3 DNA using the aforementioned method.

### Chromatin immunoprecipitation (ChIP)

DNA–protein cross-linkages were produced by fixing the CSCs of each group in formaldehyde solution for 10 min. Chromatin in the cells was broken into fragments by sonication. The cells were centrifuged at 12,000 × *g* for 10 min at 4 °C. The supernatant was then collected and divided into two tubes, to which NC rabbit IgG (ab109489, 1:300, Abcam) and E2F1 antibody (Upstate Biotech, VA, USA) were added and incubated at 4 °C overnight. The DNA–protein complexes were immunoprecipitated by Protein Agarose/Sepharose and centrifuged at 12,000 × *g* for 5 min, and the supernatant was discarded. The nonspecific complexes were washed and then cross-linked at 65 °C overnight. The DNA fragments were subsequently collected using phenol/chloroform. Primers targeting site 2 of E2F1 and the HMGB3 DNA promoter (forward: 5′-GCAGCGAGATCCGAGGACTA-3′; reverse: 5′-CCAAGGGCTTTGAAGAGGTG-3′) were designed. Another primer pair was designed distal from the HMGB3 DNA promoter region as the NC of the site 2 primer (forward: 5′-TTTTGGCTTTGTTGTTGCTGTT-3′; reverse: 5′-GCTGAAGTGGGAAGACTGGTTG-3′). The purified DNA fragment served as a model for amplification and contained site 2 primers and distal primers (control), after which RT-qPCR was performed to verify whether site 2 of the *HMGB3* gene was the binding site of E2F1.

After LINC00319 was silenced, samples containing purified DNA fragment were subjected to deep sequencing following the aforementioned steps. The sh-NC group served as the control, and the enrichment of E2F1 after binding with the HMGB3 promoter at site 2 was examined by site 2 primers.

### Cell Counting Kit-8 (CCK-8) assay

CSCs in the logarithmic growth phase were detached and seeded into a 96-well plate at 3000 cells per well. Serum-free DMEM containing 10 ng/mL basic fibroblast growth factor (bFGF), 20 ng/mL epidermal growth factor (EGF), 5 μg/mL insulin, and penicillin–streptomycin solution (100 U/mL penicillin and 100 μg/mL streptomycin) was added to each well. The blank group was treated with the culture medium only and contained no cells. The medium was replaced according to its color. A total of 10 μL CCK-8 was added at 0, 24, 48, and 72 h after seeding and incubated with the sample in the incubator for 2 h. The optical density value was evaluated at 450 nm using a microplate reader (BioTek Instruments Inc., VT, USA). The average value was determined by measuring the triplicate wells, which was then used to analyze cell growth.

### Transwell invasion assay

Extracellular matrix (ECM) gel was stored at 4 °C overnight, and the serum-free medium was diluted to 1 mg/mL at a ratio of 1:9 the next day. The polycarbonate membrane in each well of the 24-well Transwell apical chamber was coated with 40 μL ECM and incubated at 37 °C in an incubator with 5% CO_2_ for 5 h to polymerize the ECM into a gel. Next, 70 μL DMEM was added to each chamber and incubated at 37 °C for 0.5 h to allow the ECM to appropriately hydrate. The medium was then removed, after which the CSCs were serum starved for 24 h, followed by detachment, centrifugation, immunoprecipitation, and resuspension in FBS-free DMEM. The final density of the cells was 2.5 × 10^5^ cells/mL. A total of 0.2 mL cell suspension was added to the apical chamber where the basement membrane was hydrated. A total of 700 μL of precooled DMEM containing 10% FBS was added to the basolateral chamber, and the plates were cultured in an incubator at 37 °C with 5% CO_2_ under saturated humidity for a period of 24 h. The cells in the upper chamber and apical side of the membrane were removed using wet cotton swabs. The samples were then fixed in methanol for 30 min, stained with 0.1% crystal violet for 20 min, washed under running water, reverse placed, dried naturally, and observed under an inverted microscope to capture pictures. A total of five visual fields (×400) were randomly selected. The cells that were found to have invaded through the membrane were calculated and reported as the average value.

### Sphere-forming assay in vitro

CSCs were detached, centrifuged, washed once with PBS, and resuspended in FBS-free DMEM (1:1) containing 10 ng/mL bFGF, 20 ng/mL EGF, 5 μg/mL insulin, B27, and penicillin–streptomycin solution (penicillin 100 U/mL and streptomycin 100 μg/mL). The cell density was then adjusted to 1 × 10^5^ cells/mL. A total of 1 mL of the sample was added to an ultra-low adsorption culture bottle (25 cm^2^), followed by the addition of the above medium (8 mL/bottle). The cells were subsequently incubated at 37 °C with 5% CO_2_. The medium was replaced according to its color. After 1 week, five fields were selected randomly to calculate the number of spheres with a diameter ≥40 μm, after which the sphere formation rate was calculated.

### Xenograft tumor in nude mice

A total of 140 BALA/C nude mice of either sex (age: 6–8 weeks, weight: 17–22 g, Laboratory Animal Center, Zhengzhou University, Zhengzhou, Henan, China) were fed under controlled specific pathogen-free conditions. A total of 80 nude mice were grouped as follows: TU177 (5 × 10^3^), CSCs (5 × 10^3^), TU177 (1 × 10^4^), TU177 (1 × 10^4^), TU177 (5 × 10^4^), CSCs (5 × 10^4^), TU177 (1 × 10^5^), and CSCs (1 × 10^5^) (ten mice each). The cell suspensions were injected into the mice, and the tumorigenicity rate after 2 months was determined.

The following plasmids were delivered into the remaining 60 mice: oe-LINC00319, sh-LINC00319, sh-HMGB3, sh-E2F1, oe-E2F1, and their corresponding controls. A total of 1 × 10^5^ CSCs were subcutaneously injected into mice accordingly. The volume was subsequently evaluated on the tenth day after plasmid injection. The nude mice were then euthanized via carbon dioxide asphyxiation. The volume was calculated as *V* = (*A* × *B*^2^)/2 (*A*, long diameter; *B*, short diameter; mm^3^). A graph showing the average volume at each time point was made.

### Statistical analysis

All data were analyzed by SPSS 21.0 (IBM Corp., Armonk, NY, USA). Measurement data are expressed as the means ± standard deviation. Comparisons between LSCC tissues and adjacent tissues were evaluated using a paired *t* test. Data analysis between two groups were analyzed using an independent-sample *t* test. Data analysis among multiple groups were analyzed by one-way analysis of variance (ANOVA), followed by Tukey’s post hoc test. Data comparison at different time points were analyzed by repeated-measures ANOVA. *p* < 0.05 was considered to be indicative of a statistically significant difference.

## Results

### LINC00319 is overexpressed in LSCC and is associated with poor prognosis

**Fig. 1:**
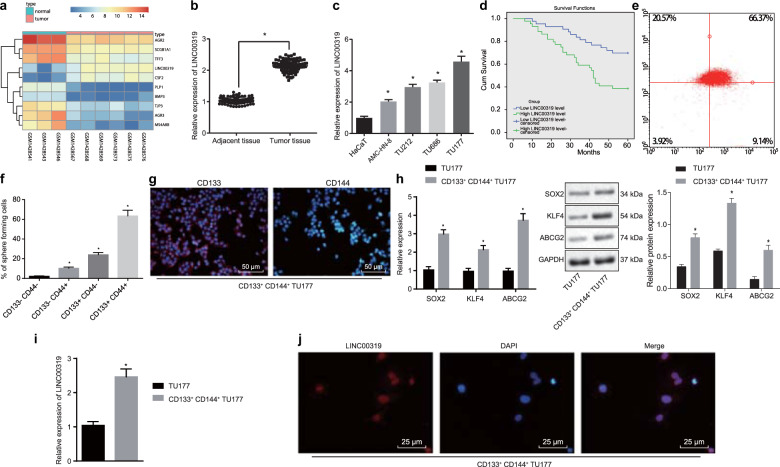
LINC00319 is overexpressed in LSCC tissues and CSCs. **a** The expression level of LINC00319 in the LSCC-related microarray dataset GSE59102 was detected. **b** RT-qPCR was used to determine the expression of LINC00319 in LSCC tissues and adjacent tissues. *p < 0.05 vs. adjacent tissue. **c** RT-qPCR was used to determine the expression of LINC00319 in four LSCC cell lines (TU177, AMC-HN-8, TU212, and TU686) and the human immortalized epidermal cell line HaCaT. *p < 0.05 vs. the HaCaT cell line. **d** Kaplan–Meier survival curve analysis was performed to analyze the correlation between LINC00319 expression and the survival rate of patients with LSCC. **e** CD133^+^CD144^+^ TU177 cells were selected from TU177 cells by flow cytometry. **f** An in vitro sphere-forming assay was used to detect the self-renewal ability of the TU177, AMC-HN-8, TU212, TU686, and HaCaT cell lines. *p < 0.05 vs. CD133^−^CD144^−^ cells. **g** The subcellular localization of CD133 and CD144 in CD133^+^CD144^+^ TU177 cells was identified by immunofluorescence (×200). **h** The expression of stem cell markers (SOX2, KLF4, and ABCG2) was determined by RT-qPCR and Western blot analysis. *p < 0.05 vs. the TU177 cells. **i** The expression of LINC00319 in T177 cells and CD133^+^CD144^+^ TU177 cells was determined by RT-qPCR^.^ *p < 0.05 vs. TU177 cells. **j** The subcellular localization of LINC00319 was identified by RNA-FISH (×400). All data were presented as the mean ± standard deviation. Comparison between the LSCC tissues and the adjacent normal tissue was analyzed by paired *t* test. Comparisons between the other two groups were analyzed by a nonpaired *t* test. Comparisons among multiple groups were analyzed by one-way ANOVA. The experiment was repeated in triplicate times.

Data from the GSE59102 microarray dataset indicated that the expression level of LINC00319 in LSCC were markedly higher than that of the adjacent tissues (Fig. 1a). For verification purposes, RT-qPCR was conducted to determine the expression of LINC00319 in LSCC tissues as well as in adjacent tissues (Fig. 1b). Relative to the adjacent tissues, LSCC tissues showed significant upregulation of LINC00319 (*p* < 0.05). The expression profile of LINC00319 was further investigated in TU177, AMC-HN-8, TU212, and TU686 LSCC cell lines and in HaCaT cell lines by RT-qPCR (Fig. 1c). The results obtained indicated that compared with HaCaT cells, TU177, AMC-HN-8, TU212, and TU686 cell lines had significantly upregulated LINC00319 expression (*p* < 0.05); the TU177 cell line exhibited the highest LINC00319 expression. The correlation between the expression of LINC00319 and the total survival rate of patients with LSCC (*n* = 87) was investigated by Kaplan–Meier survival curve analysis (Fig. 1d), the results of which highlighted the presence of a negative correlation. Thus, it was concluded based on the results obtained that LINC00319 is highly expressed in LSCC and indicative of poor prognosis in patients with LSCC.

Next, CD133^+^CD144^+^ cells were sorted from the TU177 cells via flow cytometry (Fig. 1e). The obtained four cell lines were then subjected to sphere-forming assay (Fig. 1f), and spheres exhibiting a diameter >40 μm were scored^21^. The data indicated that the spheres comprising CD133^+^CD144^+^ TU177 cells were the largest (*p* < 0.05). Immunofluorescence assays were performed to detect the subcellular localization of CD133 and CD144 in CD133^+^CD144^+^ TU177 cells (Fig. 1g), the results of which illustrated that CD133 and CD144 were highly expressed predominately in the membrane. The expression levels of stem cell markers such as SRY-box containing gene 2 (SOX2), Kruppel like factor 4 (KLF4), and ATP-binding cassette subfamily G member 2 (ABCG2) were determined using RT-qPCR and Western blot analysis methods. The results obtained indicated that compared with that in TU177 cells, the expression of SOX2, KLF4, and ABCG2 was significantly increased in CD133^+^CD144^+^ TU177 cells (*p* < 0.05) (Fig. 1h). This evidence led us to infer that the stem cells among the LSCC TU177 cell population were successfully screened out by flow cytometry. To further evaluate the tumorigenic ability of the stem cells, CD133^+^CD144^+^ TU177 and TU177 cells were diluted and injected into nude mice to establish tumor xenografts (Table S2). The results suggested that among the mice injected with CD133^+^CD144^+^ TU177 cells, the tumorigenesis rate was higher than that of mice injected with TU177 cells (*p* < 0.05), further highlighting the success of isolating CSCs from TU177 cells. Next, further investigation into the expression of LINC00319 in CSCs was performed using RT-qPCR (Fig. 1i), the results of which revealed high LINC00319 expression in CSCs relative to that of TU177 cells. RNA-FISH assays were performed to determine the subcellular localization of LINC00319 in CSCs (Fig. 1j), with a significantly greater level of LINC00319 localized in the nucleus of CSCs. These findings demonstrate that LINC00319 expression is highly upregulated in CSCs.

### LINC00319 promotes tumor growth of LSCC

Our results provide evidence supporting the overexpression of LINC00319 in CSCs in LSCC (Fig. 1). Hence, we set out to ascertain the effects of aberrant LINC00319 expression on the biological functions of CSCs in LSCC.

**Fig. 2:**
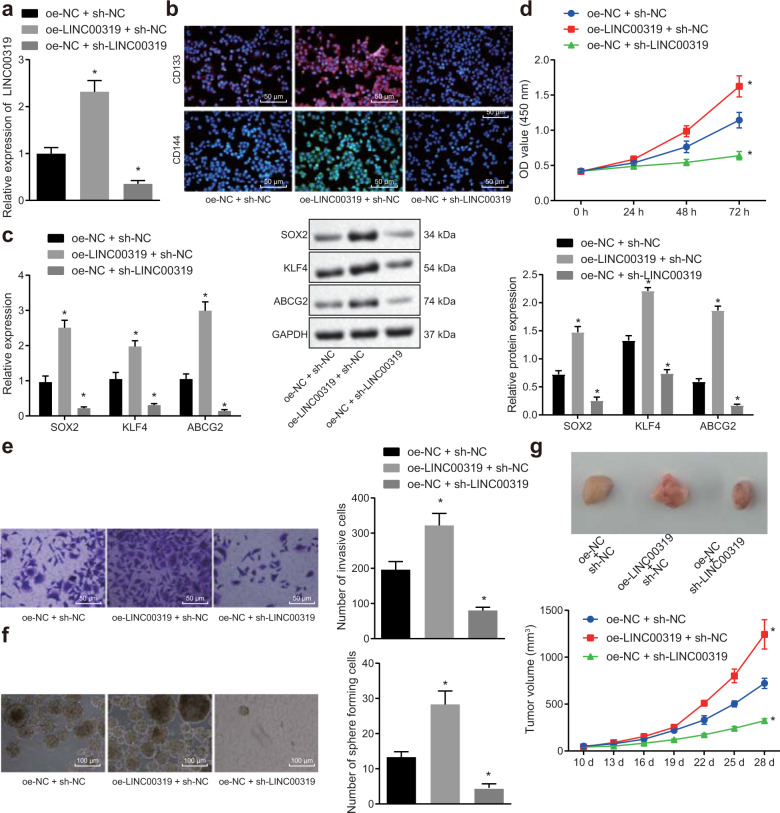
LINC00319 promotes LSCC tumor growth. **a** The transfection efficiency as indicated by the relative expression of LINC00319 in TU177 stem cells as determined by RT-qPCR. **b** The expression of CD133 and CD144 in CD133^+^CD144^+^ TU177 cells identified by immunofluorescence (×200). **c** The expression of stem cell markers (SOX2, KLF4, and ABCG2) as determined by RT-qPCR and Western blot analysis. **d** The proliferation of CSCs detected by the CCK-8 assay. **e** The invasion of CSCs detected by the Transwell assay (×200). **f** The in vitro self-renewal ability in CSCs detected by the sphere-forming assay (×100). **g** Tumorigenic ability as indicated by the tumor volume of xenograft tumors in nude mice (*n* = 10). *p < 0.05 vs. the oe-NC + sh-NC group (cells or mice treated with oe-NC + sh-NC). All data are presented as the means ± standard deviation. Comparisons among multiple groups were analyzed by one-way ANOVA. Comparisons regarding cell proliferation and tumor volume at different time points were analyzed by repeated-measures ANOVA. Cell experiments were independently repeated three times.

The transfection efficiency of LINC00319 was determined by RT-qPCR (Fig. 2a), the results of which revealed that the transfection efficiency of LINC00319 overexpression or silencing met the requirements for conducting subsequent experiments (*p* < 0.05). The localization of the overexpressed or silenced LINC00319 in TU177 stem cells was identified using an RNA-FISH assay, the results of which are depicted in Fig. S1a. Our findings indicated that in cells with overexpression of LINC00319, LINC00319 was principally localized in the nucleus, as indicated by the strongest degree of brightness, which was significantly higher than that in oe-NC + sh-NC cells. The oe-NC + sh-LINC00319 group exhibited decreased brightness compared with that of the oe-NC + sh-NC group. The aforementioned findings suggested that LINC00319 was successfully overexpressed or knocked down, indicating that exogenously overexpressed LINC00319 obviously entered the nucleus. Data were obtained indicating that CD133 and CD144 are both highly expressed in the cell membrane of CD133^+^CD144^+^ CSCs by immunofluorescence (Fig. 2b). In addition, overexpression of LINC00319 increased the expression of CD133 and CD144, while silencing LINC00319 led to a decrease in the expression of CD133 and CD144. The expression of stem cell markers (SOX2, KLF4, and ABCG2) was then measured by RT-qPCR and Western blot analysis (Fig. 2c). The results revealed that upregulation of LINC00319 elicited an increase in the expression of stem cell markers, while silencing LINC00319 led to the opposite trend (*p* < 0.05). The effects of LINC00319 on the proliferation, invasion, and self-renewal abilities of CSCs were assessed by CCK-8, Transwell, and sphere-forming assays (Fig. 2d–f). The results illustrated that overexpression of LINC00319 could promote proliferation, invasion, and self-renewal ability in vitro, all of which could be reversed by silencing LINC00319 (*p* < 0.05). In addition, the effect of LINC00319 on tumorigenic ability was investigated using xenograft tumors in nude mice, the results of which indicated that overexpression of LINC00319 could enhance the tumorigenic abilities of the CSCs, while silencing LINC00319 suppressed their tumorigenic ability (*p* < 0.05) (Fig. 2g). Altogether, the aforementioned results suggest that LINC00319 acts as a tumor promoter in LSCC.

### LINC00319 increases the expression of HMGB3 by recruiting E2F1

**Fig. 3:**
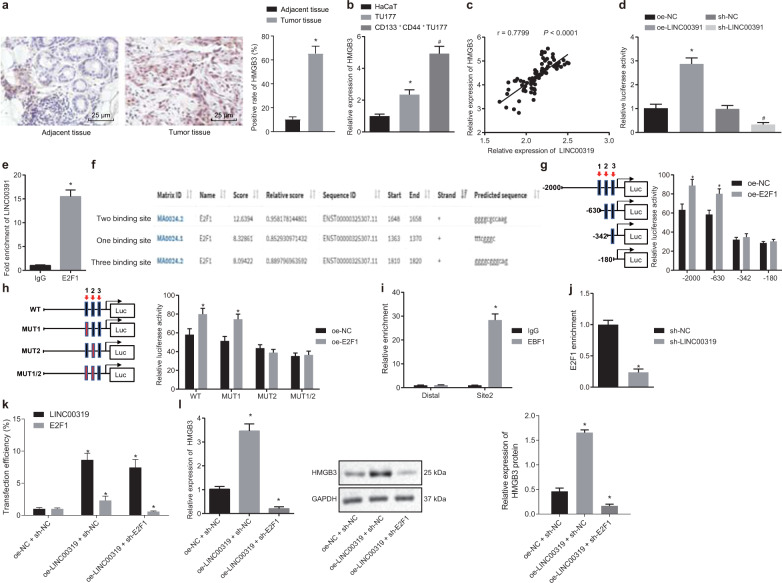
LINC00319 increases the expression of HMGB3 via E2F1. **a** Immunohistochemistry was used to determine the expression of HMGB3 in LSCC and adjacent tissues (×400), *p < 0.05 vs. adjacent tissue. **b** RT-qPCR was employed to determine the expression of HMGB3 in cells, *p < 0.05 vs. HaCaT cells, ^#^*p* < 0.05 vs. TU177 cells. **c** Correlation analysis between LINC00319 and HMGB3 expression in LSCC tissues (*n* = 87). **d** The dual-luciferase reporter gene assay was used to detect the influence of LINC00319 on the activity of the HMGB3 promoter, *p < 0.05 vs. the oe-NC group (cells treated with oe-NC), ^#^*p* < 0.05 vs. the sh-NC group (cells treated with sh-NC). **e** RIP confirmed that LINC00319 could bind to E2F1, *p < 0.05 vs. the IgG group. **f** The three potential binding sites between E2F1 and HMGB3 DNA according to website prediction. **g**, **h** The specific binding site was identified by the dual-luciferase reporter gene assay, *p < 0.05 vs. the oe-NC group. **i** The binding ability of E2F1 to HMGB3 on site 2 as detected by ChIP assay, *p < 0.05 vs. the IgG group. **j** The relative enrichment of E2F1 as determined by ChIP assay in CSC with silenced LINC00319 expression, *p < 0.05 vs. the sh-NC group. **k** The transfection efficiency of LINC00319 and E2F1 as detected by RT-qPCR, *p < 0.05 vs. the oe-NC + sh-NC group (cells transfected with oe-NC and sh-NC). **l** RT-qPCR and Western blot analysis were employed to determine the expression of HMGB3 in the presence of LINC00319 overexpression and E2F1 silencing. *p < 0.05 vs. the oe-NC + sh-NC group. All data are presented as the means ± standard deviation. Comparisons between the LSCC and adjacent tissues (*n* = 87) were analyzed by paired *t* test, and comparisons between the other two groups were analyzed by nonpaired *t* test. Comparisons among multiple groups were analyzed by one-way ANOVA. Cell experiments were repeated in triplicate.

Predictions from the LncMAP database (http://www.bio-bigdata.com/LncMAP/index.jsp) suggested that LINC00319 regulates the expression of the *HMGB3* gene by recruiting E2F1. To explore the underlying relationship of LINC00319, E2F1, and HMGB3 in LSCC, RT-qPCR and immunohistochemistry were conducted (Fig. 3a, b), demonstrating that HMGB3 is highly expressed in LSCC tissues and cells as indicated by its higher levels in CSCs than in normal cells (*p* < 0.05). The correlation between LINC00319 and HMGB3 in LSCC tissues (*n* = 87) was then analyzed, and the results revealed a positive correlation (*p* < 0.05) (Fig. 3c).

We subsequently turned our attention to verifying the associated regulatory mechanism. A dual-luciferase reporter assay was conducted by co-transfecting oe-NC, oe-LINC00319, sh-NC, and sh-LINC00319 with HMGB3-2Kb luciferase reporter vectors to determine the influence of LINC00319 on HMGB3 (Fig. 3d). The results revealed that HMGB3 promoter activity was strengthened in LSCC cells with oe-LINC0031, but weakened in cells with sh-LINC00319 (*p* < 0.05), suggesting that LINC00319 could positively regulate HMGB3 expression. Next, a RIP assay was performed, which confirmed that E2F1 could significantly bind to a greater number of LINC00319 molecules relative to that identified in the IgG group (Fig. 3e). A dual-luciferase reporter gene assay was performed to further verify the direct binding between LINC00319 and E2F1 using cells transfected with oe-NC, oe-LINC00319, sh-NC, and sh-LINC00319, as illustrated in Fig. S1b. The results revealed a relatively higher luciferase intensity in the presence of LINC00319 overexpression, while reduced luciferase intensity was observed in the cells with LINC00319 silencing, indicating that LINC00319 directly binds to E2F1. For the identification of specific binding sites, UCSC (http://genome.ucsc.edu/) and JASPAR (http://jaspar.genereg.net/) were introduced for analysis, the results of which indicated three binding sites where E2F1 was most likely to bind to the HMGB3 promoter (Fig. 3f). We subsequently identified site 2 as the most likely specific binding site and co-transfected truncated or MUT HMGB3 reporter plasmids with E2F1 into CSCs in order to conduct a dual-luciferase reporter gene assay (Fig. 3g, h). A ChIP assay was then performed to determine the binding ability of E2F1 on site 2 in the HMGB3 promoter region (Fig. 3i), the result of which indicated that the interaction of E2F1 with site 2 was capable of producing more amplification products than its interaction with the distal promoter (*p* < 0.05), with no significant difference identified in the IgG group (*p* > 0.05). This further demonstrated that site 2 (ggggcgccaag) in the promoter region of HMGB3 was the likely binding site of E2F1. Next, exploration was conducted to further elucidate the effect of LINC00319 on the regulation of E2F1 and HMGB3 DNA by means of a ChIP assay (Fig. 3j). The results obtained demonstrated that E2F1 enrichment was significantly diminished in the presence of sh-LINC00319 (*p* < 0.05).

To further ascertain the involvement of E2F1 in the regulatory mechanism of LINC00319 on HMGB3, the cells were treated with oe-LINC00319 and sh-E2F1 along with their respective NC (Fig. 3k). RT-qPCR and Western blot analysis were conducted again to quantify HMGB3 expression in order to verify the regulatory mechanism among LINC00319, E2F1, and HMGB3 (Fig. 3l). The results demonstrated that ectopic LINC00319 overexpression increased HMGB3 expression, which could be reversed by silencing E2F1 (*p* < 0.05). These findings provide clarification and insight into the regulatory role of LINC00319 on the expression of HMGB3, which is achieved specifically via the recruitment of E2F1.

### LINC00319 promotes tumor growth in LSCC by upregulating HMGB3

Since the regulatory mechanism between LINC00319 and HMGB3 has yet to be uncovered, our exploratory emphasis was shifted to the effects of the underlying mechanism on the biological characteristics of CSCs in LSCC via overexpression of LINC0319 and silencing of HMGB3.

**Fig. 4:**
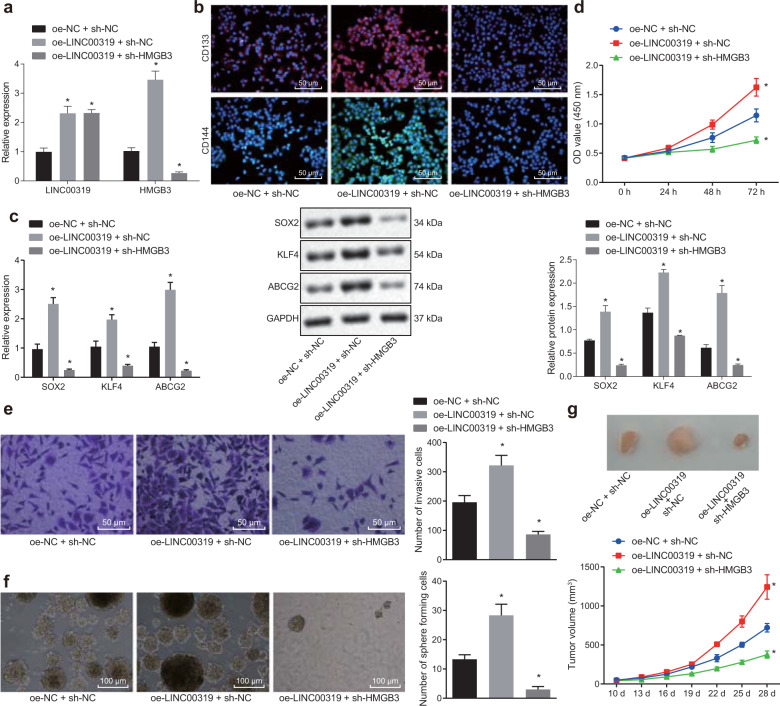
LINC00319 promotes LSCC tumor growth by upregulating HMGB3 expression. **a** RT-qPCR was used to determine the expression of LINC00319 and HMGB3 in TU177 stem cells from each group. **b** The expression of CD133 and CD144 in CD133^+^CD144^+^ TU177 cells was determined by immunofluorescence (×200). **c** The expression of stem cell markers in TU177 stem cells was determined by RT-qPCR and Western blot analysis. **d** The CCK-8 assay was used to detect cell proliferation. **e** Transwell assays were used to detect cell invasion (×200). **f** The in vitro self-renewal ability of CSCs was detected by sphere-forming assays (×100). **g** The tumorigenic ability of CSCs was investigated in xenograft tumors in nude mice (*n* = 10). *p < 0.05 vs. the oe-NC + sh-NC group (cells or mice treated with oe-NC + sh-NC). All data are presented as the means ± standard deviation. Comparisons among multiple groups were analyzed by one-way ANOVA. Comparisons at different time points were analyzed by repeated-measures ANOVA. Cell experiments were independently repeated three times.

RT-qPCR was employed to detect the plasmid transfection efficiency in cells after transduction (Fig. 4a). The data obtained revealed that the transfection had reached the standard required for initiation of the following experiments (*p* < 0.05). The expression of CD133 and CD144 in CD133^+^CD144^+^ TU177 cells was determined by immunofluorescence (Fig. 4b). The expression of CD133 and CD144 was higher in the membrane of CD133^+^CD144^+^ TU177 cells. LINC00319 overexpression was associated with higher expression of these proteins, whereas silencing HMGB3 reversed the aforementioned effects (*p* < 0.05). RT-qPCR and Western blot analyses were then employed to detect the expression of SOX2, KLF4, and ABCG2 (Fig. 4c), with their expression markedly elevated in the cells overexpressing LINC00319 and significantly decreased in the presence of sh-HMGB3 (*p* < 0.05). CCK-8, Transwell, and sphere-forming assays were used to evaluate the effects of LINC00319 on the proliferation, invasion, and self-renewal abilities of CSCs in vitro (Fig. 4d–f). The results revealed that cell proliferation, invasion, and self-renewal abilities were significantly enhanced following LINC00319 overexpression, while these changes were notably reversed in cells with HMGB3 silencing (*p* < 0.05). The xenograft tumors in nude mice also exhibited the same trend (*p* < 0.05, Fig. 4g).

The aforementioned results provided evidence verifying that LINC00319 can promote the biological characteristics of CSCs in LSCC by increasing the expression of HMGB3.

### LINC00319 is involved in the biological characteristics of LSCC cells by promoting HMGB3 via E2F1

**Fig. 5:**
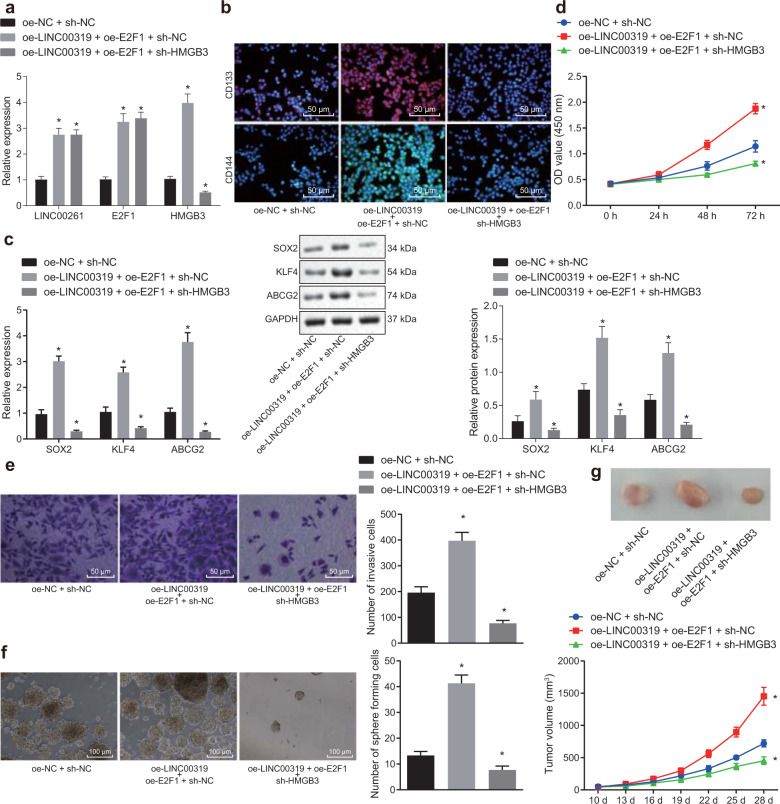
LINC00319 promotes tumor growth by increasing HMGB3 expression via E2F1. **a** RT-qPCR was used to determine the expression of LINC00319, E2F1, and HMGB3 in TU177 stem cells from each group. **b** The expression of CD133 and CD144 in CD133^+^CD144^+^ TU177 cells was examined by immunofluorescence (×200). **c** The expression of SOX2, KLF4, and ABCG2 in TU177 stem cells was determined by RT-qPCR and Western blot analysis. **d** CCK-8 was used to detect cell proliferation. **e** Transwell assays were used to detect cell invasion (×200). **f** The in vitro self-renewal ability of CSCs was detected by sphere-forming assays (×100). **g** The tumorigenic ability of CSCs was investigated in xenograft tumors in nude mice (*n* = 10). *p < 0.05 vs. the oe-NC + sh-NC group (cells or mice treated with oe-NC + sh-NC). All data are measurement data and are presented as the means ± standard deviation. Comparisons among multiple groups were analyzed by one-way ANOVA. Comparisons at different time points were analyzed by repeated-measures ANOVA. The cell experiments were independently repeated three times.

Finally, the effect of the mechanism involving LINC00319, E2F1, and HMGB3 on the biological characteristics of LSCC was evaluated in cells transduced with plasmids containing oe-LINC00319, oe-E2F1, and sh-HMGB3. RT-qPCR was employed to determine the plasmid transfection efficiency in cells with LINC00319 overexpression, E2F1 overexpression, and HMGB3 silencing (Fig. 5a), and the results in each group were confirmed to have met the standard required for further investigation (*p* < 0.05). The expression of CD133 and CD144 in CD133^+^CD144^+^ TU177 cells was determined by immunofluorescence analysis (Fig. 5b). Higher expression of CD133 and CD144 was detected in the membrane and in CD133^+^CD144^+^ TU177 cells transfected with oe-LINC00319, while the presence of sh-HMGB3 partially diminished the expression of CD133 and CD144 (*p* < 0.05). In addition, our results indicated that the expression of SOX2, KLF4, and ABCG2 notably increased in the cells overexpressing LINC00319 and overexpressing E2F1, while significant decreases were detected in the cells with silenced HMGB3 (*p* < 0.05) (Fig. 5c). Finally, CCK-8, Transwell, and sphere-forming assays were carried out to show that cell proliferation, invasion, and self-renewal were significantly enhanced in the cells overexpressing LINC00319 and overexpressing E2F1, while markedly reduced levels were detected in the cells with silenced HMGB3 (*p* < 0.05) (Fig. 5d–f). The xenograft tumor results in nude mice further confirmed the aforementioned trend (Fig. 5g).

Taken together, these findings provide evidence elucidating the promoting effects of LINC00319 on the biological characteristics of CSCs in LSCC, accomplished by increasing the expression of HMGB3 via recruitment of E2F1.

## Discussion

Laryngeal cancer represents one of the most prevalent cancers worldwide and is the most common cause of all malignant tumors of the larynx^2^. The diagnosis of LSCC as well as staging of this disease requires a complex series of evaluations; however, accumulating evidence continues to highlight the potential of molecular approaches for the diagnosis and treatment of LSCC^22,23^. In the current study, we set out to explore the regulatory mechanism of LINC00319 on the biological processes and characteristics of LSCC, with our observations suggesting that overexpression of LINC00319 helps to promote the proliferative, invasive, and self-renewal abilities of CSCs in LSCC, ultimately contributing to tumorigenesis.

In the current study, evidence was obtained proving that LINC00319 is expressed at higher levels in LSCC cells and tissues than in noncancerous cells and tissues, with high levels of LINC00319 expression associated with poor prognosis. LINC00319 expression has been identified to be upregulated in cutaneous squamous cell carcinoma with studies suggesting its relationship with poor prognosis is due to its ability to promote cell proliferation and invasion;^8^ a similar trend was observed in the data of the current study. In addition, in the setting of lung cancers, reports have shown that LINC00319 is highly expressed in cell lines and is associated with cellular progression, including cell proliferation and invasion, by reducing the expression of microRNA-32^24^. In addition, although LINC00319 has been found to be highly expressed in LSCC, numerous other lncRNAs have also been reported to have elevated levels in LSCC, many of which contribute to the regulation of tumorigenesis. LINC00460 has been reported to exhibit marked increases in LSCC and consequently promotes cellular proliferation and invasion by sponging miR-145^9^. Studies have reported that lncRNA H19 is upregulated in LSCC and regulates cell proliferation, migration, and invasion by cooperating with miR-148a-3p^25^. Our results indicated that increased LINC00319 expression promoted the proliferation, invasion, and self-renewal abilities of CSCs in LSCC and facilitated tumor growth. These findings suggest that LINC00319 plays a crucial role in LSCC treatment as an oncogene and should be considered a clinical target validated by more studies.

The HMGB family is a group of highly conserved chromatin-associated proteins and consists of four members: HMGB1, HMGB2, HMGB3, and HMGB4^26^. In our study, we identified that by recruiting E2F1, LINC00319 was able to regulate the function of HMGB3. In gastric cancer, the mechanism of HMGB3 has been investigated, whereby knockdown of HMGB3 has been speculated to be responsible for the suppression of the cell cycle and tumor growth in gastric cancer^10^. In liver cancer, evidence has been presented suggesting that overexpression of lncRNA TP73-AS1 promotes hepatocellular carcinoma cell proliferation partly due to its role in increasing the expression levels of HMGB1^27^. Recent literature has indicated that the expression of HMGB2 is elevated in various human cancers, suggesting that the small heterodimer partner represses HMGB2 by recruiting or repressing E2F1 activity ^17^. Thus, the evidence suggests that HMGB3 plays an oncogenic role in promoting tumor growth in various types of cancers.

Our data suggest that LINC00319 regulates LSCC by recruiting E2F1. Previous reports have highlighted that lncRNA MALAT plays an activating role in the CDK4/E2F1 signaling pathway of breast cancer^28^. A previous study collectively proposed that overexpression or knockdown of lncRNA-HIT can promote or reduce cell proliferation in non-small cell lung cancer, respectively, largely by regulating E2F1 in the promoter regions of its target genes^29^. The aforementioned studies suggest that lncRNAs influence other types of cancers, particularly from the perspective of cell progression and their interaction with E2F1. Interestingly, we investigated the relationship among LINC00319 and other factors and found that LINC00319 is directly involved in the regulation of E2F1 at recognized sites; moreover, a crucial reason for the oncogenic function of LINC00319 in LSCC is based upon its interaction with E2F1 and its subsequent regulation of the expression of HMGB3. These studies provide further indication of the notable potential of LINC00319 to bind to E2F1 and interfere with the progression of LSCC.

**Fig. 6:**
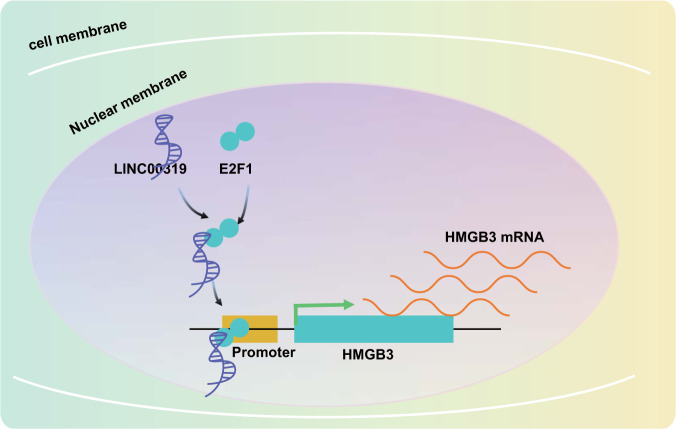
Schematic map concerning the functional role of LINC00319 in LSCC. LINC00319 induces cell proliferation, invasion, and self-renewal ability in vitro and tumor growth in vivo in LSCC by increasing HMGB3 expression via recruitment of E2F1.

Taken together, the key findings of the current study provide evidence demonstrating the promoting mechanism of LINC00319 on cell proliferation, invasion, and self-renewal ability in vitro and tumor growth in vivo in LSCC by increasing HMGB3 expression via recruitment of E2F1 (Fig. 6). However, further investigations emphasizing the downstream targets of HMGB3 to elucidate the complete signaling pathway regulated by LINC00319 are required. In addition, considering the mechanism we have described, our findings may provide new insights into the prognostic or therapeutic treatment of LSCC.

## Supplementary information

Supplemental information
