# Supplementary material for: LINC00319 promotes cancer stem cell-like properties in laryngeal squamous cell carcinoma via E2F1-mediated upregulation of HMGB3
Source: Exp Mol Med. 2021 Aug 18;53(8):1218–28. doi: 10.1038/s12276-021-00647-2 (PMC8417254; doi:10.1038/s12276-021-00647-2)
Supplement: Supplementary file 1 — Supplemental information [file 12276_2021_647_MOESM1_ESM.docx]

**Table S1** Primer sequences of genes for RT-qPCR

| Genes | Primer sequences |
| --- | --- |
| LINC00319 | F: 5'-GGAAGCCGGATAAGCACCTC-3' |
|  | R: 5'-GCTACGCTGCAGTCACAAAC-3' |
| HMGB3 | F: 5'-GTTCAGCTTCAGGCCAAGTG-3' |
|  | R: 5'-GGCATAAGCGGACATCTTGC-3' |
| GAPDH | F: 5'-TCAGCAATGCCTCCTGCAC-3' |
|  | R: 5'-TCTGGGTGGCAGTGATGGC-3' |
| SOX2 | F: 5'-GGGAAGGGAGGGAGGGACTA-3' |
|  | R: 5'-TGCAAAGCTCCTACCGTTCC-3' |
| KLF4 | F: 5'-ATGCTCACCCCACCTTCTTC-3' |
|  | R: 5'-TTCTCACCTGTGTGGGTTCG-3' |
| ABCG2 | F: 5'-CGCACAGAGCAAAGCCATTT-3' |
|  | R: 5'-GCAAGGGGCTAGAAGAAGGG-3' |

**Note:** RT-qPCR, reverse transcription quantitative polymerase chain reaction; F, forward; R, reverse; HMGB3, high-mobility group box 3; GAPDH, glyceraldehyde-3-phosphate dehydrogenase; SOX2, SRY-box containing gene 2; KLF4, Krüppel-like factor4; ABCG2, ATP-binding cassette subfamily G member 2.

**Table S2** Tumorigenicity of CD133^+^CD144^+^TU177 cells

| Injected cells | TU177 | CD133^+^CD144^+^TU177 |
| --- | --- | --- |
| 5 × 10^3^ | 0/12 | 8/12 |
| 1 × 10^4^ | 0/12 | 8/12 |
| 5 × 10^4^ | 4/12 | 12/12 |
| 1 × 10^5^ | 8/12 | 12/12 |
| Total | 12/48(25%) | 40/48(83. 3%) |

**
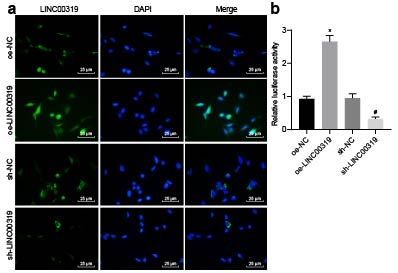
**

**Fig. S1** Assessment of location of LINC00319 and binding between LINC00319 and E2F1. a, Localization of overexpressed/silenced LINC00319 in TU177 stem cells detected by RNA-FISH (400 ×). b, Binding between LINC00319 and E2F1 detected by dual-luciferase reporter gene assay. * *p* < 0.05 *vs.* the oe-NC group (cells treated with oe-NC). # *p* < 0.05 *vs.* the sh-NC group (cells treated with sh-NC).
